# Subduction intraslab-interface fault interactions in the 2022 *M*_w_ 6.4 Ferndale, California, earthquake sequence

**DOI:** 10.1126/sciadv.adl1226

**Published:** 2024-03-06

**Authors:** David R. Shelly, Dara E. Goldberg, Kathryn Z. Materna, Robert J. Skoumal, Jeanne L. Hardebeck, Clara E. Yoon, William L. Yeck, Paul S. Earle

**Affiliations:** ^1^ U.S. Geological Survey, Golden, CO 80401, USA.; ^2^ U.S. Geological Survey, Moffett Field, CA 94035, USA.; ^3^ U.S. Geological Survey, Pasadena, CA 91106, USA.

## Abstract

The Mendocino triple junction—the intersection of the Pacific, North American, and Gorda plates—activates a collection of disparate faults that reconcile Cascadia subduction with San Andreas transform motion. The 20 December 2022 *M*_w_ 6.4 Ferndale, California, earthquake occurred within this complex zone as strike-slip faulting within the subducting Gorda slab. Here, we analyze the seismic and geodetic signatures of the mainshock and aftershock sequence to illuminate its role within complex tectonic surroundings. We find aftershocks on varied fault structures within the uppermost Gorda slab, yet seismicity on the subduction interface itself was notably absent. Nevertheless, we identify small but coherent postseismic deformation that is well modeled by aseismic slip on this interface, likely triggered by stresses generated at the updip limit of coseismic rupture. This sequence demonstrates the potential for interactions between intra-slab earthquakes and slip on the subduction megathrust, highlighting the need to consider this and other subduction zones as coupled systems of interacting faults.

## Teaser

The 2022 *M*_w_ 6.4 Ferndale earthquake ruptured in the Gorda slab but triggered postseismic slip on the subduction megathrust fault.

## INTRODUCTION

**Fig. 1.**
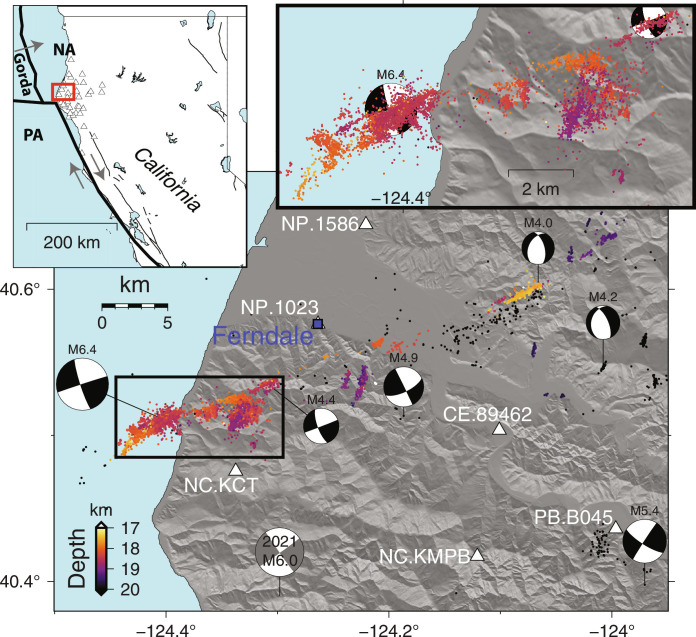
Map of 2022 *M*_w_ 6.4 Ferndale earthquake and its aftershocks. Black beachballs show available NCSN moment tensor solutions for the mainshock and its aftershocks through 20 January 2023. Detected and relocated aftershocks are plotted, color-coded by depth (black dots show those events at depth > 20 km). Seismic stations with names are shown by white triangles. Gray beachball shows the moment tensor solution for the 2021 onshore, intraslab *M*_w_ 6.0 event as determined by Yeck *et al.* (*3*). Inset in top left shows location of main map view (red box) and seismic stations used in this study (triangles). Plate boundaries are shown by bold black lines (PA, Pacific plate; NA, North American plate) (*58*), with plate motions schematically indicated by gray arrows. Inset in top right shows zoomed view of the most active western zone of seismicity (shown as black box in the main map). Events are color-coded by depth as in the main map.

The Mendocino triple junction accommodates the abrupt transition of western US tectonics from Cascadia subduction in the north to San Andreas transform motion in the south. Here, the Pacific, North American, and Gorda plates collide (Fig. 1), generating high rates of seismicity. Faulting in this zone occurs with a variety of orientations, with both seismic and aseismic movement (*1*–*3*).

A collection of evidence suggests that the deformation required by this motion is largely accommodated by internal deformation of the Gorda plate, which is younger and weaker than the bordering Pacific or North American plates. For this reason, the Gorda plate is sometimes referred to as the “Gorda deformation zone” (*4*, *5*) or even “the so-called Gorda plate” (*6*). This internal deformation is accompanied by a high rate of seismicity within the southern Gorda plate itself, much of it occurring along former spreading ridge normal faults rotated and reactivated as northeast-striking, left-lateral faults (*6*–*8*). Slip on these faults partially accommodates north-south shortening within the Gorda plate, imposed by the northward motion of the Pacific plate, with the subducted Gorda slab possibly fragmenting and flowing into the slabless window created east of the northern San Andreas Fault (*7*). Near the triple junction, internal Gorda seismicity occurs together with seismicity along the right-lateral Mendocino Transform fault (*1*) and seismicity within the overriding North American plate including the 1992 *M* (magnitude) 7.1 Cape Mendocino earthquake (*9*–*11*). Ruptures have also been observed to interact among these faulting regimes; the 1992 reverse-faulting event exhibited large strike-slip aftershocks within the Gorda slab (*9*, *11*), while a sequence on 20 December 2021 included two temporally overlapping *M* ~ 6 earthquakes, one offshore on the Mendocino transform fault and the other ~11 s later and ~30 km northeast onshore within the subducting Gorda slab (Fig. 1) (*3*).

On 20 December 2022, 1 year after the multi-fault 2021 rupture, the *M*_w_ (moment magnitude) 6.4 Ferndale earthquake ruptured within the subducted Gorda slab near the coastline, ~15 km northwest of the onshore 2021 event. The 2022 mainshock caused two fatalities and extensive damage in the epicentral region, including approximately 150 homes damaged or destroyed (https://earthquake.usgs.gov/earthquakes/eventpage/nc73821036/impact, last accessed 22 September 2023). This event also generated a robust aftershock sequence that extended ~40 km along an east-northeast (ENE) trend. This distribution was broadly consistent with one of the nodal planes of the mainshock moment tensor, implying a primarily left-lateral strike-slip rupture (Fig. 1).

In this study, we investigated the kinematics and dynamics of faulting in this earthquake sequence to ascertain its role in the broader tectonics of the Mendocino triple junction and the Cascadia subduction zone. In particular, we aimed to understand the relationship between this rupture and the Cascadia megathrust fault, which is capable of generating *M* 9–class earthquakes (*12*). Toward that goal, we performed integrated earthquake detection, relocation, and focal mechanism analysis for the first month of the aftershock sequence, leveraging earthquakes routinely detected and located by the Northern California Seismic Network (NCSN) as waveform templates. We combined this analysis with modeling of the mainshock coseismic slip using both seismic and geodetic data. Last, we identified and modeled a subtle postseismic transient.

## RESULTS

### Relocations and focal mechanisms

Detected and relocated Ferndale aftershock seismicity reveals a clear ENE-striking trend in map view that mostly bounds the northern extent of aftershock activity (Fig. 1). This trend supports predominantly left-lateral strike-slip motion in the mainshock, consistent with one of the moment tensor nodal planes. Most aftershocks are concentrated within a relatively narrow depth range of 17 to 20 km. Aftershocks observed south of the main ENE trend appear to align on smaller structures mostly striking approximately north, which were not apparent in the routine catalog (fig. S1). Multiple zones within the rupture generated prolific aftershocks during the first month following the *M*_w_ 6.4 mainshock, with no obvious spatial migration (fig. S2).

Moment tensors of the four largest aftershocks near this main trend (*M*_w_ 4.0 to 4.9) indicate a mix of faulting orientations, with the two closest to the mainshock epicenter showing strike-slip motion, while two farther east indicate predominantly extensional faulting (Fig. 1). These normal-faulting events are also located somewhat deeper than most of the aftershocks, with relocated depths of 22 to 24 km. A fifth large aftershock, an *M*_w_ 5.4 strike-slip event, is located farther southeast of the rupture, offset ~20 km horizontally from the main trend and even deeper, at ~30-km depth.

**Fig. 2.**
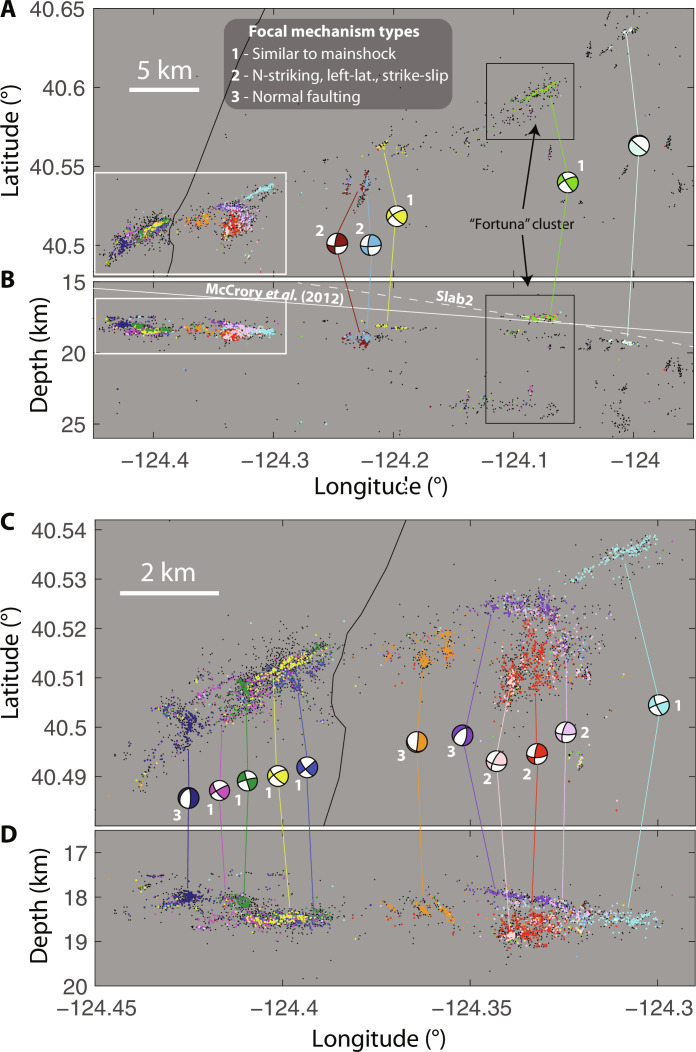
Detected and relocated Ferndale aftershock seismicity, 19 December 2022 through 20 January 2023. Events with correlation-based focal mechanisms are color-coded by mechanism group. Corresponding mechanism solutions are shown in map view, with a line connecting to primary zone of occurrence. Numbers accompanying mechanisms indicate qualitative categorization (see "Relocations and focal mechanisms") according to legend at top (the eastern-most mechanism is poorly constrained and not categorized). Events without mechanisms are shown in black. (**A**) Map view, and (**B**) cross section. White dashed line shows the Slab2 (*13*) model, while the solid white line shows the model of McCrory *et al.* (*10*) for the subduction interface. Black boxes show the area where the Fortuna (*23*), and equivalent C2 (*15*) clusters were observed with long-term activity before the 2022 earthquake. White boxes in (A) and (B) show the extents of zoomed views in parts (C) and (D), respectively. (**C**) Zoomed map view of dense western zone of seismicity. (**D**) Corresponding zoomed cross section.

Figure 2 shows map view and cross-sectional views of aftershock seismicity, along with focal mechanism solutions obtained using correlation-based relative polarities and *S/P* amplitude ratios. On the basis of the correspondence with existing plate interface models (*10*, *13*), converted seismic phases (*14*, *15*), and tomography studies (*9*), which approximately track the shallow eastward dip of the upper extent of seismicity, we infer that seismicity was likely concentrated within the upper ~2 to 4 km of the Gorda slab. The strike orientations of the focal mechanism nodal planes are mostly consistent with the trends defined by the earthquake locations. Because of limitations in focal sphere coverage, the nodal plane dips of some solutions are poorly constrained (fig. S3), and unmodeled complex ray paths [e.g., (*15*)] may increase misfit. Despite these caveats, focal mechanisms are broadly consistent with three categories, as labeled in Fig. 2: (1) strike-slip mechanisms with orientation similar to the mainshock moment tensor, consistent with ENE-striking, left lateral faulting; (2) mechanisms with an approximately north-striking nodal plane, dominantly left-lateral strike-slip with perhaps a component of oblique normal faulting, mostly south of the main rupture plane; and (3) normal faulting mechanisms, with a north-striking nodal plane, again mostly south of the main rupture. Notably absent is any evidence for thrust-type aftershocks on the subduction interface itself, despite the apparent proximity of faulting within the uppermost Gorda slab. Also notable is the coexistence of both ENE-striking and north-striking left-lateral mechanisms. This could imply suboptimally oriented faulting on one or both orientations. Alternatively, this could indicate a stress state that is highly variable in space or one that was locally altered by slip in the mainshock (*16*).

### Coseismic slip modeling

**Fig. 3.**
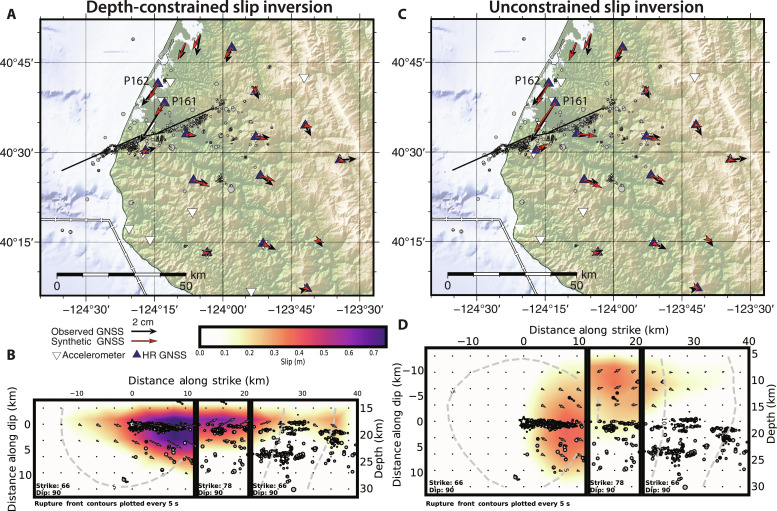
Coseismic rupture models. (**A**) Depth-constrained slip inversion and (**B**) associated slip distribution from limiting slip to >15-km depth (approximate depth of the slab surface in this area). Note the overall good correspondence between observed (black) and modeled (red) GNSS vectors, with the notable exception of station P161. The star in each plot indicates the relocated mainshock hypocenter. Gray dashed lines in the cross sections show the modeled rupture front at 5-s intervals. Relocated aftershocks from this study are plotted as black circles, with diameter scaled linearly with magnitude (*M* 0 to 5.4). (**C**) Slip inversion without depth constraint and (**D**) associated slip distribution. The depth-constrained model in (A) and (B) corresponds well with aftershock seismicity, making it our preferred solution.

Global Navigation Satellite Systems (GNSS) data seem to paint a picture of coseismic rupture that contrasts with the relocated aftershocks. Figure 3 shows observed and modeled coseismic displacements for two different slip models: one in which slip is constrained to >15-km depth (roughly the depth of the top of the Gorda slab) and one in which the depth of slip is unconstrained. Although most data are well fit by both models (figs. S4 and S5), the modeled displacement at station P161 in the depth-constrained model is much smaller than observed. Station P161 has by far the largest recorded coseismic surface displacement of 3 cm, although station P162, located 6 km to the north, shows a smaller coseismic displacement of 1.8 cm. Without imposing depth constraints on slip, these data lead to a best-fitting model that has substantial slip extending as shallow as ~5 km. However, slip to such shallow levels is difficult to reconcile with the lack of aftershocks observed from these depths.

On the basis of the spatial distribution of aftershocks, the most reasonable inference would be that the *M*_w_ 6.4 mainshock ruptured the uppermost part of the subducting slab. The reason for such high rates of seismicity near the slab surface itself is unclear, but clusters of persistent seismicity are active within this zone even before this sequence, demonstrating that this is a longer-term feature of seismicity in this area. Multichannel active-source seismic data have shown that the uppermost Gorda Plate is pervasively faulted offshore (*7*, *17*), which likely facilitates hydration of this crust from seawater infiltration (*18*). This may lead to areas with elevated fluid pressure in the uppermost Gorda plate as it subducts and slab temperatures begin to rise (*19*), which could result in locally concentrated deformation and seismicity.

How do we reconcile this discrepancy between the shallow slip apparently required by the P161 offset and the deeper slip implied by the aftershock seismicity? We argue that the lack of shallow aftershocks is likely a stronger constraint than the single large displacement at station P161. For example, station P161 is located at the edge of the Eel River Basin, which might affect its coseismic deformation response (*20*); odd behavior was also noted in modeling of a nearby *M*_w_ 6.5 event that occurred offshore in 2010 (*21*). Furthermore, the apparent slip distribution may be affected by the required simplification of the fault geometry for modeling. Although aftershocks might not be expected within the deepest portions of the upper plate, due to elevated temperatures that might inhibit brittle failure (*22*), the lack of aftershocks becomes increasingly hard to explain in scenarios where slip extends into the upper overriding crust, particularly to depths < 10 km. For example, the nearby 1992 *M* 7.1 Cape Mendocino earthquake generated an active aftershock sequence in the upper plate at ~10-km depth (*9*–*11*). Therefore, we argue that the collective evidence most strongly supports the model where coseismic slip is mostly confined within the slab.

If slip reached the upper surface of the slab, then it would have the potential to interact with the subduction megathrust fault. We can consider two end-member scenarios of behavior for the megathrust. In one extreme where the subduction interface remains locked, slip propagating to the upper surface of the slab would likely continue into the upper plate. In the other extreme where the interface slips freely, then coseismic slip that reaches the surface of the slab would likely transition to the interface, setting up a geometry whereby the Gorda slab effectively subducts south of the rupture and remains fixed north of the rupture (under the assumption that existing stresses make a reversal of subduction to the north unlikely), with the differential motion accommodated along the main left-lateral strike-slip fault.

We evaluated the possibility of coseismic slip on the subduction interface by testing a finite fault model that included a shallowly dipping fault, approximating this interface. However, the resultant inversion did not resolve slip occurring coseismically on this subduction interface fault; instead, the inversion showed coseismic rupture concentrated on the left-lateral strike-slip fault, consistent with the mainshock moment tensor. Therefore, the models presented here include only the strike-slip fault.

### Postseismic slip modeling

**Fig. 4.**
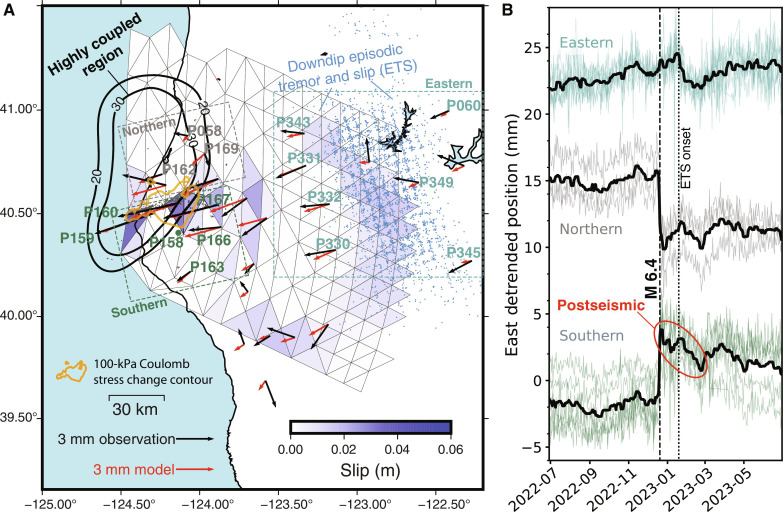
Postseismic deformation. (**A**) Slip from inversion of observed GNSS displacements between 21 December 2022 and 28 February 2023. Slip amounts are shown in blue, with aftershocks of the 2022 Ferndale earthquake from this study shown in gray. Black lines show contours of interseismic locking on the Cascadia subduction interface, in millimeters per year, from Materna *et al.* (*26*). The 2-month displacements are well fit by slip of up to ~4 cm on the Cascadia subduction interface, mostly south of the 2022 Ferndale rupture. The +100-kPa Coulomb stress contour computed from the depth-constrained coseismic rupture model is shown in orange (fig. S6). A typical downdip episodic tremor and slip (ETS) event also took place during this time window; this slip created westward displacements on the eastern stations and can be recognized as ~2 cm of inverted slip in the deeper part of the subduction interface. Station groups plotted in (**B**) are labeled. Tremor epicenters [https://pnsn.org/tremor, last accessed 27 July 2023; (*59*)] associated with this downdip slip are shown as blue dots. (B) Stacked GNSS timeseries, east component, for selected groups of stations. Stations are collected into northern, southern, and eastern groups, as color-coded in (A). Only stations labeled in (A) are plotted in (B). Daily solutions are plotted as dots, superimposed on each other for each group. The median-filtered average displacements for each group are plotted as bold black lines to highlight coherent behavior. Timing of the *M* 6.4 mainshock and approximate onset of the downdip ETS event are indicated.

**Fig. 5.**
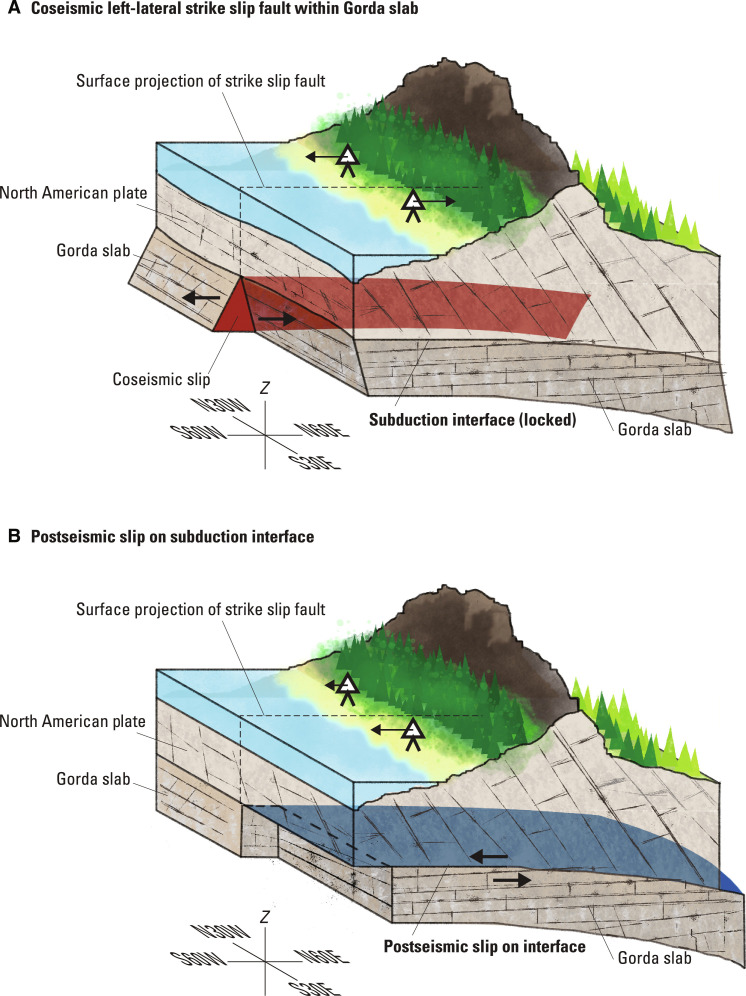
Cartoon of coseismic and postseismic slip interpretation. (**A**) Coseismic left-lateral strike-slip motion on a vertical plane within the subducting Gorda slab (pink-shaded region). Triangles with arrows depict qualitative coseismic surface displacements. (**B**) Postseismic slip on the subduction interface, primarily south of the rupture (blue shaded region). Triangles with arrows depict qualitative postseismic surface displacements, reversing the sense of coseismic motion south of the rupture.

While we did not find evidence for coseismic slip on the subduction interface, we did observe apparent postseismic westward motion at several sites south of the rupture (Fig. 4), opposite in direction to the coseismic motion, occurring over ~2 months following the mainshock. Although the GNSS displacements are small (~3 mm), they are coherent in the zone south of the rupture. Modeling shows that they are well explained by up to ~4 cm of postseismic convergence on the subduction interface, primarily on the south side of the mainshock rupture (Fig. 4). The Coulomb failure stress imparted on the interface by the preferred (depth-constrained) coseismic slip model is dominantly encouraging of such slip, exceeding 200 kPa near the mainshock rupture (fig. S6). Thus, we propose that coseismic slip occurred primarily on an approximately vertical left-lateral strike-slip fault within the upper portion of the slab, and postseismic slip occurred primarily on the subduction interface south of the coseismic rupture (Fig. 5). Any afterslip on the left-lateral ENE-trending strike-slip coseismic fault would create westward surface displacements north of the mainshock rupture that would add constructively to westward postseismic displacements generated by afterslip on the subduction interface (Fig. 5); because observed postseismic displacements north of the rupture are small, we infer that any afterslip on the coseismic fault is minor. If a small amount of afterslip occurred on the mainshock fault, then slightly higher postseismic slip on the subduction interface would be required to overcome expected eastward displacements south of the rupture and produce the observed westward motion.

## DISCUSSION

The Mendocino triple junction presents a complex intersection of faults with a history of seismic interactions among them, including in the 1992 and 2021 earthquake sequences discussed previously. The 2022 *M*_w_ 6.4 Ferndale earthquake sequence provides the latest such example, with coseismic slip within the Gorda slab likely triggering postseismic slip on the subduction interface.

The aftershock distribution of the Ferndale earthquake only hints at these broader interactions but may reflect the history of faulting and hydration within the Gorda plate. North-south compression of the young Gorda slab has been accommodated by extensive faulting and perhaps breakup of the Gorda slab as it subducts and begins to fill the slabless window east of the northern terminus of the San Andreas fault (*7*). These faults likely facilitated hydration of the uppermost Gorda crust before subduction. Resultant high fluid pressures may contribute to the concentration of aftershocks within the uppermost Gorda slab at depths of 17 to 20 km.

Previously faulted and hydrated areas may also form localized zones of weakness with long-lived clusters of seismicity over a period of at least several years (*15*, *23*). Although these clusters locate near the subduction interface, evidence from focal mechanisms, locations, and waveform characteristics indicates that they likely occur within the Gorda slab rather than on the interface itself. Gong and McGuire (*15*) identified five seismicity clusters preceding the 2022 Ferndale sequence by many years. They inferred these clusters occurred mostly in the uppermost Gorda slab based on phases reflected from the slab Moho. Their seismicity cluster “C2” seems to correspond with the “Fortuna” cluster examined by Alongi *et al.* (*23*), who noted increased seismic activity in concert with an apparent change in coupling on the subduction interface. Notably, the aftershock sequence of the 2022 *M*_w_ 6.4 rupture exhibits high rates of seismicity within the same zone as this previously studied cluster, including a linear zone of strike-slip events concentrated at 17- to 18-km depth (Fig. 2 and fig. S2) and an *M*_w_ 4.0 normal-faulting event located at nearly 24-km depth (Fig. 1). This suggests that long-lived conditions of this zone contribute to its seismogenesis, such as possible high fluid pressure.

A further puzzle posed by aftershock activity near this “Fortuna cluster” is that the pronounced linear zone of Ferndale aftershock seismicity (light green events in Fig. 2) is located ~1 to 2 km shallower than the gently dipping trend of earthquakes within the surrounding clusters. Although the absolute depth difference is small and well within uncertainties of existing plate models, this seismicity feature is persistent in aftershock relocations for this sequence. Multiple interpretations of this apparent geometric complexity near the surface of the Gorda slab are plausible. One possible explanation is that this feature coincides with a subducted seamount (*24*), with seismicity perhaps occurring within fluid-rich sediments entrained behind the seamount as it subducts (*25*) or near the apex of the seamount itself. Alternatively, this feature could represent subduction of a horst structure, similar to those seen in the Gorda plate offshore using active-source seismic imaging (*7*, *17*). High fluid pressures within this zone might help to explain the corresponding long-lived locus of persistent seismicity, both preceding the 2022 earthquake and during its aftershock sequence. Resonance within subducted sediments might also explain the delayed high-amplitude *S*-wave arrivals previously observed from this seismicity cluster, arrivals that were absent from earthquakes occurring several kilometers beneath this cluster, deeper within the slab (*15*). We also note that, based on interpretations of active seismic imaging, Gulick *et al.* (*17*) inferred 1 to 2 km of sediment subducting with the Gorda slab near the deformation front; plausibly, the shallower seismicity could be occurring near the top of the sediment layer with slightly deeper surrounding seismicity near the top of the underlying slab crust. The long-lived nature of this cluster before the 2022 sequences makes it less likely that it simply represents impingement of the 2022 rupture into the upper plate.

The lack of thrust-mechanism aftershocks coincident with postseismic slip on the subduction interface suggests that this part of the interface exhibits rate-strengthening behavior, inhibiting dynamic (coseismic) failure but amenable to postseismic creep. Long-term geodetic modeling shows that the immediate area around the *M*_w_ 6.4 rupture is mostly coupled before the rupture but transitions to largely creeping farther south (Fig. 4) (*26*). In the transition, the interface may be kinematically coupled because of physical locking farther north, rather than frictionally locked (*27*). This along-strike transition from locked to creeping would itself strain the slab, encouraging left-lateral motion like that observed in the *M*_w_ 6.4 event. A similar mechanism may have contributed to the 2020 *M* 7.6 Sand Point, Alaska earthquake, a strike-slip event in the subducting slab near the Shumagin gap (*28*).

A major tremor episode initiated farther downdip in mid-January 2023, ~3 weeks following the mainshock (Fig. 4 and fig. S4). Although this might suggest that aseismic slip on the subduction interface propagated downdip into the tremor zone over the weeks following the rupture, two factors make this unlikely. First, modeling of postseismic slip (Fig. 4) shows a large gap in slip between the coseismic rupture area and the deeper slow slip zone. Second, the timing of the tremor episode in January 2023 was consistent with the history of activity over the past several years (fig. S3). Therefore, the January 2023 tremor and slip episode was likely unrelated to the Ferndale rupture and its postseismic deformation.

Interactions between intraslab earthquakes and slip on the subduction interface, while not commonly reported, have been previously inferred. For example, although they lacked local data, Abercrombie *et al.* (*29*) inferred similar behavior in the 4 June 2000 *M*_w_ 7.9 earthquake south of Sumatra. On the basis of teleseismic waveform modeling, they proposed that the earthquake initiated as left-lateral strike-slip within the slab, before transitioning to thrust motion on the subduction interface. In Northeast Japan, Uchida *et al.* (*30*) analyzed repeating earthquake sequences and inferred that slow slip occurred on the plate interface around the time of three large (*M* 6.9+) earthquakes within the underlying slab. Intriguingly, they concluded that slip was not only postseismic but also preseismic, initiating days to months before the earthquakes, reflecting stress transfer between the subduction interface and the slab itself.

Multiple examples of dominantly aseismic slip on the subduction interface preceding a megathrust earthquake have also been reported, including for the 2011 *M*_w_ 9.0 Tohoku-Oki earthquake (*31*) and the 2014 *M*_w_ 8.1 Iquique, Chile, earthquake (*32*–*34*). These examples reinforce the coexistence of slow and dynamic slip behaviors exhibited in subduction zones, as well as the potential for transitions between these behaviors (*33*).

The 2022 Ferndale earthquake sequence presents an intriguing example of interactions among faults in the Mendocino triple junction. Additional studies and investments in geophysical networks would help to further resolve the dynamics of this complex zone. Seismic and geodetic observational networks have improved substantially in recent years, offering a glimpse of this complexity, yet questions remain. For example, our preferred model confines coseismic slip primarily within the subducting Gorda slab, corresponding with the observed aftershock distribution, yet is unable to reproduce the relatively large GNSS offset observed at station P161. In addition, the zone of slightly shallower aftershock seismicity corresponding with the previously observed Fortuna seismic cluster suggests complexity in the subducting slab geometry, but the origin of this complexity is now unknown.

Subduction zones host the largest earthquakes on the planet. In combination with previously observed interactions between slow and fast slip and between intraslab and interface slip, observations of the 2022 Ferndale sequence emphasize the importance of considering subduction zones as coupled systems of interacting faults. In Cascadia, seismicity rates are high near the Mendocino triple junction compared to relative quiescence farther north within the subduction zone. Given the demonstrated potential for fault interactions, this could increase the likelihood of faulting within the triple junction as a potential nucleation site for future Cascadia megathrust events.

## MATERIALS AND METHODS

### Earthquake detection and relocation

To create a high-resolution earthquake catalog for analysis, we worked to both expand the number of detected earthquakes and to improve their location precision. To achieve this, we applied an integrated event detection and relocation approach, following the method described by Shelly *et al.* (*35*). We used 309 earthquakes routinely cataloged by the NCSN as waveform templates by searching within a 50-km radius of a point near the center of the aftershock sequence (40.575° latitude, −124.126° longitude) for the period spanning 19 December 2022 through 20 January 2023 (fig. S1).

Waveform templates were formed separately for *P* and *S* wave packets, using a window length of 2.5 s (*P*) or 4.0 s (*S*), beginning 0.3 s before the phase arrival. We used phase data from NCSN to determine arrival times; if no *S* phase was available, then we approximated the *S* arrival time using the NCSN *P* arrival time and origin time with a *P*-to-*S* velocity ratio (*V_p_/V_s_*) of 1.73. If needed, *P* templates were truncated to avoid overlapping with *S* templates. All data were bandpass filtered 2 to 10 Hz, resampled to 100 Hz (if necessary), and templates were scanned through continuous seismic data using the correlation algorithm of Beaucé *et al.* (*36*).

We processed data in daily batches from 19 December 2022 through 20 January 2023. We declared a provisional detection when the summed correlation across all channels exceeded eight times the median absolute deviation (MAD) for that day. Subsequently, we examined individual channels for those with an absolute value correlation exceeding seven times the daily MAD for that channel, up to a maximum lag of 0.5 s for *P* and 0.85 s for *S*. To achieve subsample precision, we used three-point quadratic interpolation of the correlation function. In cases with at least four channels exceeding this threshold, we saved the differential times for use in relocation. We enforced a minimum separation time between detected events of 4.0 s for a given template, to avoid multiple detections of the same event.

Before relocating the events, we combined detections from all templates, associating common events based on estimated origin time. We then input all correlation-derived differential times along with differential times derived from catalog phase arrival times into the hypoDD double-difference algorithm (*37*). We used the one-dimensional (1D) “MEN” (Mendocino) velocity structure model determined by Castillo and Ellsworth (*38*) for this area. After inversion, we considered events retaining at least 20 combined (*P* and *S*) differential times to be well constrained. In total, 7809 events met this criterion and are presented here.

### Event magnitudes

We retained NCSN catalog magnitudes for catalog (template) events. We computed magnitudes for newly detected events using NCSN duration magnitudes (*M*_d_) and measured amplitude ratios between the newly detected event and the template on each correlated data channel. For each template that detected a given event, a provisional magnitude was determined using the median amplitude ratio among all correlated data channels, assuming that a factor of 10 in amplitude equates to one magnitude unit difference. For events detected by multiple templates (the usual case), the final magnitude was determined using the median estimate from all templates that detected it. As the magnitudes were not a primary focus of this work, we did not attempt to calibrate magnitude scaling to match observed scaling of catalog magnitudes.

### Focal mechanisms

An important aid to interpreting the aftershocks is constraining their sense of slip; therefore, we extended the seismic analysis to estimate focal mechanisms of aftershocks. Because relatively few events are large enough to produce unambiguous *P*-wave polarities across the network, we followed the approach described by Shelly *et al.* (*39*), which can constrain relative polarities for lower signal-to-noise events. This technique uses the sign of the maximum absolute value correlation between two waveforms as a proxy for their relative polarities. We further used imputation-based estimation of missing relative polarity values (*40*) and *S/P* amplitude ratios calculated by a combination of single-component relative amplitudes with traditional three-component *S*/*P* amplitude ratios (*41*, *42*). Last, mechanisms were computed using the HASH software (*43*, *44*).

We reused correlation and amplitude measurements from our initial processing, following the approaches described by Shelly *et al.* (*39*) for correlation-derived polarities (using routine analyst-determined polarities to resolve the sign ambiguity) and by Shelly *et al.* (*41*) for single-component–derived *S/P* amplitude ratios. These data were combined with traditional *S/P* amplitude ratios. Traditional *S/P* amplitudes were computed considering the 1747 *M* ≥ 2 earthquakes in the region −124.5°E to −123.9°E longitude, 40.3°N to 40.7°N latitude, between January 2000 and March 2023. If an earthquake lacked a manually identified *S*-phase arrival, then an estimated arrival time was calculated using the source-receiver *P*-wave travel time and a *V_p_/V_s_* ratio of 1.73. We filtered the waveforms on the 34 available three-component stations between 2 and 12 Hz and integrated them to displacement. The noise levels and *P* amplitudes were determined using the Cartesian sum of the radial and vertical components. The peak amplitude in the window 2.5 to 0.5 s before the *P*-wave arrival defined the noise level. The peak amplitude in the window beginning 0.5 s before and 1.5 s after the *P-*wave arrival defined *P* amplitude. The *S* amplitude was the peak amplitude on the three-component components within 2 s following the *S*-wave arrival.

Following Skoumal *et al.* (*40*, *45*), we imputed missing polarity measurements and then categorically clustered the events into families. After calculating the reconciled polarity weights, we used the sign of the weights to convert them into categorical data. These categorical weights were represented as either a positive, negative, or missing value in an *n × p*–dimensional matrix, **X**, composed of *n* earthquakes and *p* stations. Considering only the earthquakes with ≥25 reconciled polarity weights above a threshold of 10^−10^, we imputed missing polarity data with the missForest algorithm (*46*), which used random forests (*47*) in an iterative fashion. Once complete, **X** was a complete categorical matrix containing either positive or negative values for all considered earthquake-station pairs. Using these polarity data, we then categorically clustered the measurements using the *k*-modes algorithm (*48*) into 15 families.

Station corrections for the *S/P* amplitude ratios were derived from the set of traditional *S/P* amplitude ratios, which more fully sample the focal sphere than the Ferndale sequence alone. For each of the 34 three-component stations, the distribution of log_10_(*S/P*) for that station was shifted to line up its median value with the median theoretical log_10_(*S/P*) assuming uniform sampling of the focal sphere. This shift defines the station correction. The distribution of corrected log_10_(*S/P*) observations resembles the theoretical distribution, although it is slightly narrower, indicating perhaps incomplete sampling of the focal sphere.

Composite focal mechanisms for each cluster were computed from the correlation-derived *P*-wave polarities and the station-corrected *S/P* amplitude ratios using a version of the HASH code (*43*, *44*). The HASH code was modified to weight each polarity with a weighting parameter based on the confidence of the cross-correlation polarity assignment, as described by Shelly *et al.* (*39*). The ray azimuths and takeoff angles for each earthquake-station pair were computed using the 1D MEN velocity model of Castillo and Ellsworth (*38*).

### Coseismic rupture modeling

We performed kinematic finite-fault modeling of the 2022 *M*_w_ 6.4 mainshock using the Wavelet and simulated Annealing SliP (WASP) package (*49*). WASP uses nonlinear simulated annealing in the wavelet domain to solve for slip amplitude, rake, rupture time, and rise time (*50*, *51*). We jointly inverted teleseismic broadband, local strong-motion accelerometer, and both high-rate and static GNSS data. Static offsets were estimated by the Nevada Geodetic Laboratory (*52*), and high-rate observations were processed with PRIDE PPP-AR software (*53*). The model consists of three planar fault segments, each with vertical dip and varying in strike. This model was derived to fit the general spatial distribution of the aftershock sequence, which contains a small jog and thus was not well fit by a single planar fault. We implemented Laplacian smoothing across the fault segments, allowing continuous rupture across the fault segment boundaries. We modeled two end-member rupture possibilities: (i) with rupture allowed to extend into the upper (North American) plate without any depth constraint and (ii) with rupture constrained to below 15-km depth, for better consistency with aftershock depths. Model fits to data are compared in figs. S4 and S5.

### Postseismic slip modeling

We constrained postseismic deformation transients using the Network of the Americas GNSS time series, which are processed by precise point positioning (*54*) in the ITRF2014 reference frame (*55*) and transformed into a North America–fixed realization. We then applied additional post-processing to remove secular trends, sinusoidal functions with annual and semiannual frequency and coseismic offsets. We solved for postseismic offsets between 21 December 2022 and 28 February 2023 at each station, with the initial and final estimate of station position determined by the mean of a 3-day averaging window. Last, we selected a subset of GNSS offsets by removing obvious outlier stations (those with displacements > 20 mm: P335, P794, and P156) and stations outside of the region of interest (Fig. 4). We calculated uncertainties on the displacements by taking the uncertainties on the average positions of the prior and posterior epochs and adding them in quadrature, resulting in typical horizontal uncertainties of 2 to 4 mm and typical vertical uncertainties of 10 to 14 mm.

For postseismic fault slip modeling, we assumed a Cascadia subduction geometry consisting of 237 triangular fault patches shallower than 50-km depth [the same geometry used by Materna *et al.* (*56*)]. We related slip to displacements using the heterogeneous Green’s functions from Materna *et al.* (*56*), which account for variations in elastic properties within the earth. We then solved the following equation by bounded least squares to provide the best-fitting fault slip model *m*

$$\left[\begin{array}{c} W*d\\ 0\\ 0 \end{array}\right]=\left[\begin{array}{c} W*G\\ \lambda L\\ \alpha \left(z\right)I \end{array}\right]m$$

where **G** is the Green’s matrix, **W** is the diagonal weighting matrix, *d* is a vector containing the horizontal GNSS offsets, **L** is a Laplacian smoothing matrix with strength λ, and α is the strength of minimum-norm penalty (i.e., zeroth-order Tikhonov regularization) multiplied by the identity matrix. The slip model *m* consists of a single rake-parallel component for each triangular mesh element. For the zeroth-order Tikhonov regularization, the strength of the regularization penalty increased slightly with depth *z* to suppress spurious noise in the deeper portion of the fault. We set the strength of the Laplacian smoothing and Tikhonov regularization by L-curve analysis. For the model presented in Fig. 4, the result has an average misfit of 2.9 mm.

To evaluate the causality between the mainshock rupture and the modeled postseismic deformation, we modeled associated stress changes using the formulation of Okada (*57*). We computed Coulomb and shear stress changes imposed by the depth-constrained coseismic rupture model of Fig. 3 (A and B) on receiver faults derived from the same Cascadia subduction zone geometry as the postseismic slip modeling (fig. S6). The rake of all receiver faults was set to 90° (pure reverse), the assumed coefficient of friction was 0.4, and the assumed shear modulus and Poisson’s ratio were 30 GPa and 0.25, respectively.
