# Supplementary material for: Subduction intraslab-interface fault interactions in the 2022 Mw 6.4 Ferndale, California, earthquake sequence
Source: Sci Adv. 2024 Mar 6;10(10):eadl1226. doi: 10.1126/sciadv.adl1226 (PMC10917346; doi:10.1126/sciadv.adl1226)
Supplement: Supplementary file 1 — Figs. S1 to S7 [file sciadv.adl1226_sm.pdf]

Science Advances
AAAS

Supplementary Materials for

Subduction intraslab-interface fault interactions in the 2022 M_w 6.4 Ferndale, California, earthquake sequence

David R. Shelly *et al.*

Corresponding author: David R. Shelly, dshelly@usgs.gov

Sci. Adv. **10**, eadl1226 (2024)
DOI: 10.1126/sciadv.adl1226

This PDF file includes:

Figs. S1 to S7

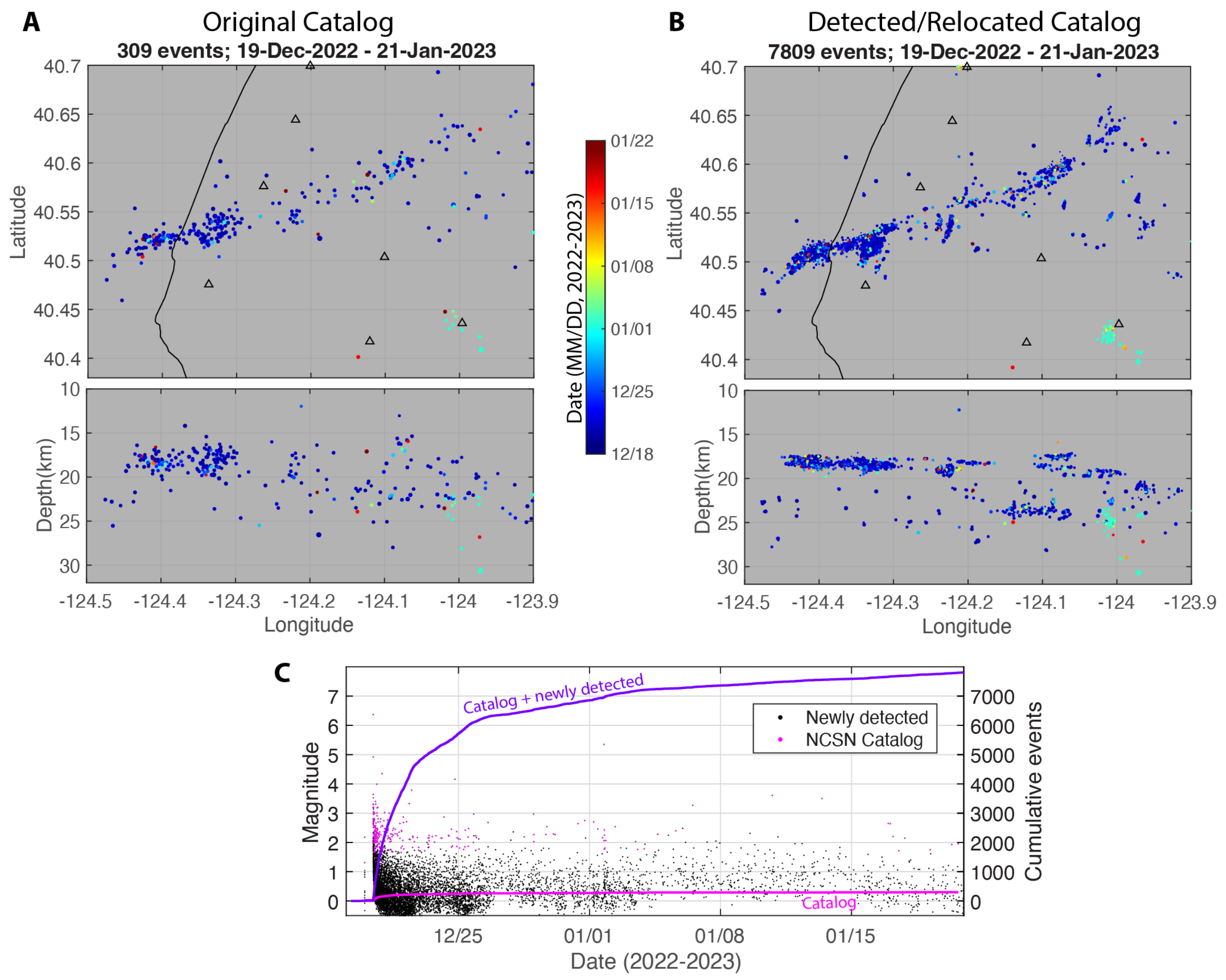

Fig. S1. Comparison between the original routine earthquake catalog and the high-resolution detected/relocated catalog produced in this study. (A) Original NCSN catalog, containing 309 events. Events are color-coded by occurrence time as indicated. **(B)** High-resolution detected/relocated catalog produced in this study. Events are color-coded by time as in (A). **(C)** Magnitude versus time plot for the original NCSN catalog (magenta dots) and the newly detected events (black dots). Cumulative number of events are shown on the right axis, with the magenta line (original NCSN catalog) and the purple line (high resolution catalog containing both original catalog events and newly detected events).

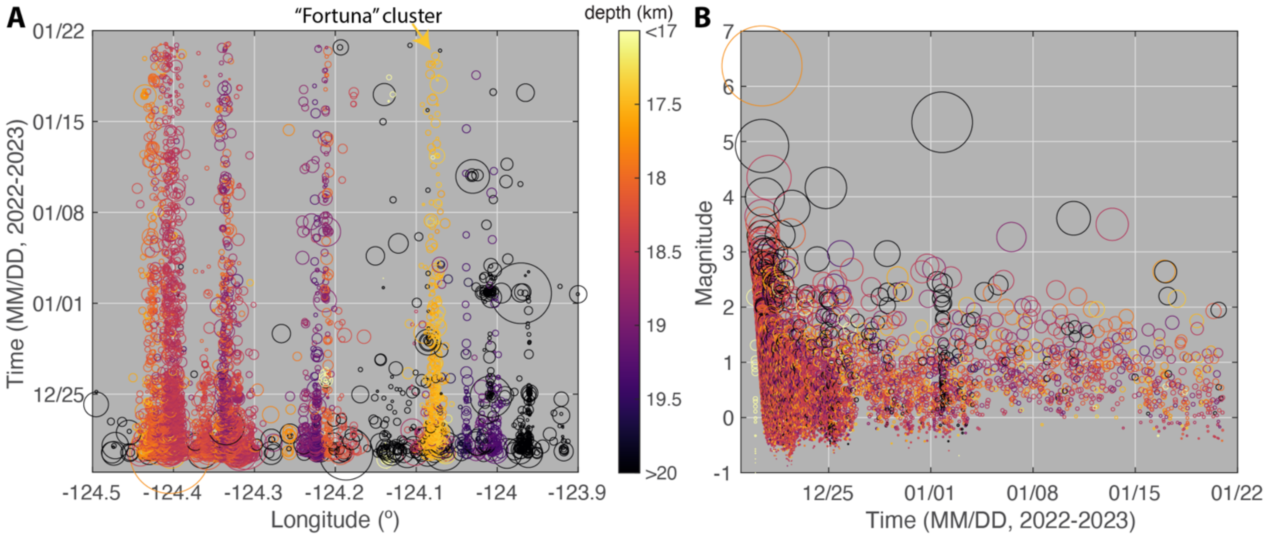

Fig. S2. Temporal evolution of the sequence. (A) Earthquake time versus longitude. Events are color-coded by depth as indicated. Circle size scales with magnitude, with sizes the same as in part (B). **(B)** Earthquake magnitude versus time. Events are color-coded by depth and circle size scales with magnitude as in (A).

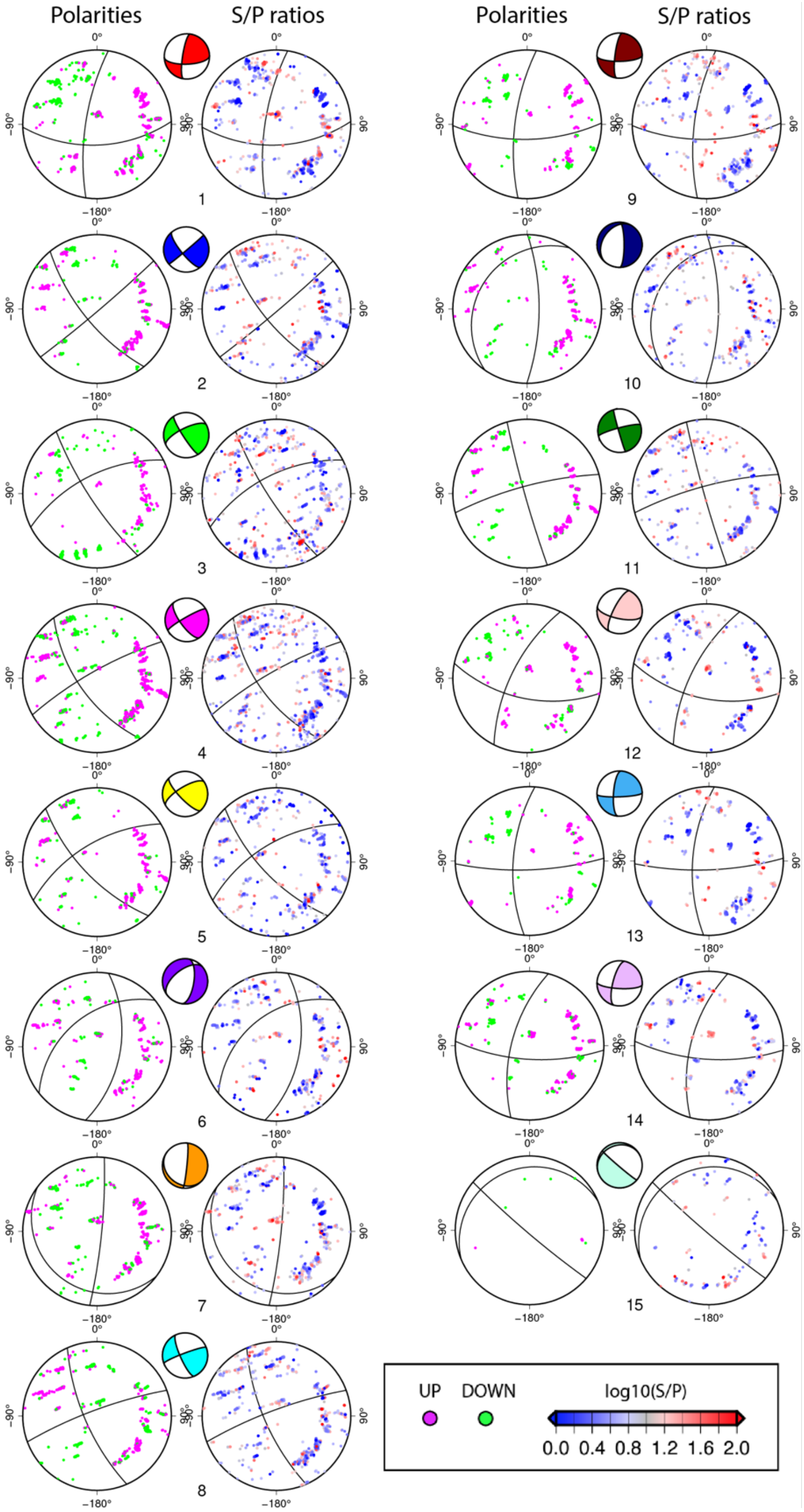

Fig. S3. **Focal mechanism solutions for each mechanism group**. Left columns show *P*-wave first-motion polarities. Right columns show *S/P* amplitude ratios (color scale in lower right). Colored mechanisms correspond with those in Figure 2.

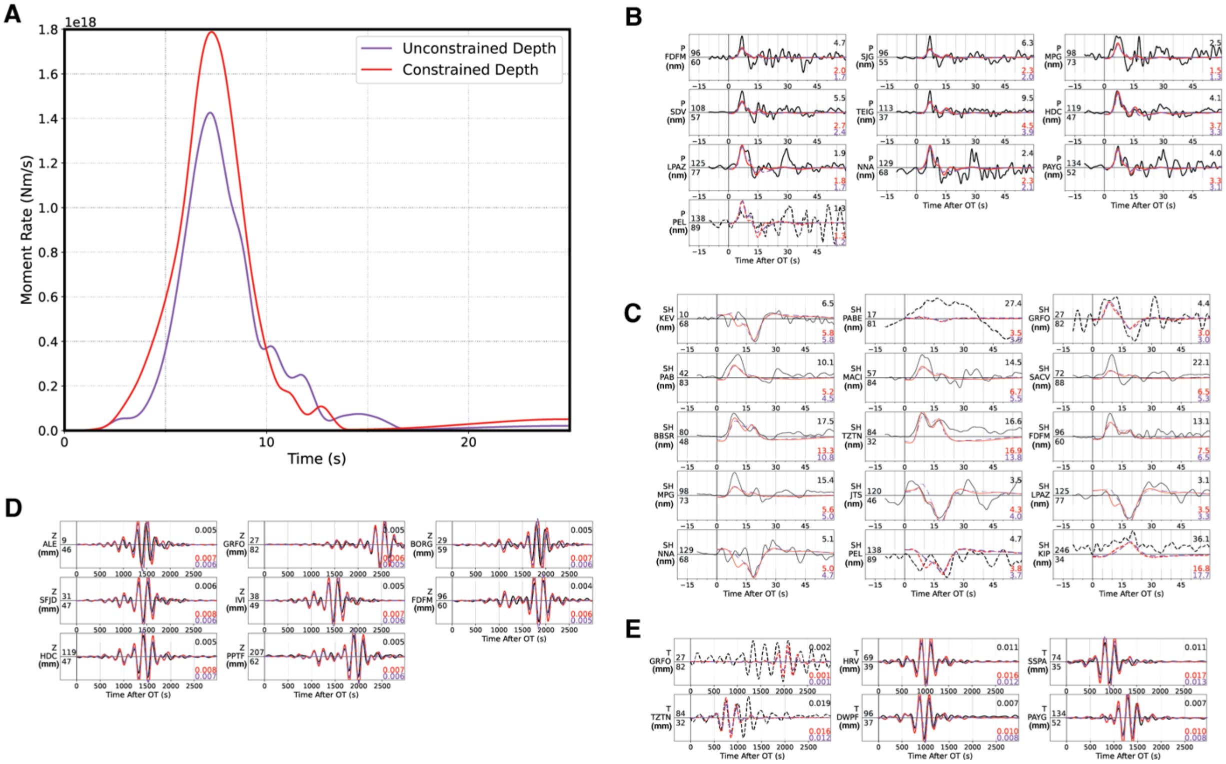

Fig. S4. Finite fault modeling results, comparing depth constrained (red) and unconstrained (purple) models. **(A)** Source time functions, **(B-E)** Observed waveforms (black), depth-constrained (red) and unconstrained (purple) models for **(B)** teleseismic P-waves, **(C)** teleseismic SH-waves, **(D)** teleseismic Rayleigh waves, and **(E)** teleseismic Love waves. In the left edge of each plot, the top number denotes the station azimuth while lower value denotes the station distance (in degrees). On the right edge of each plot, the numbers in black, red, and purple denote the maximum amplitude for the observations, depth constrained model, and unconstrained model, respectively.

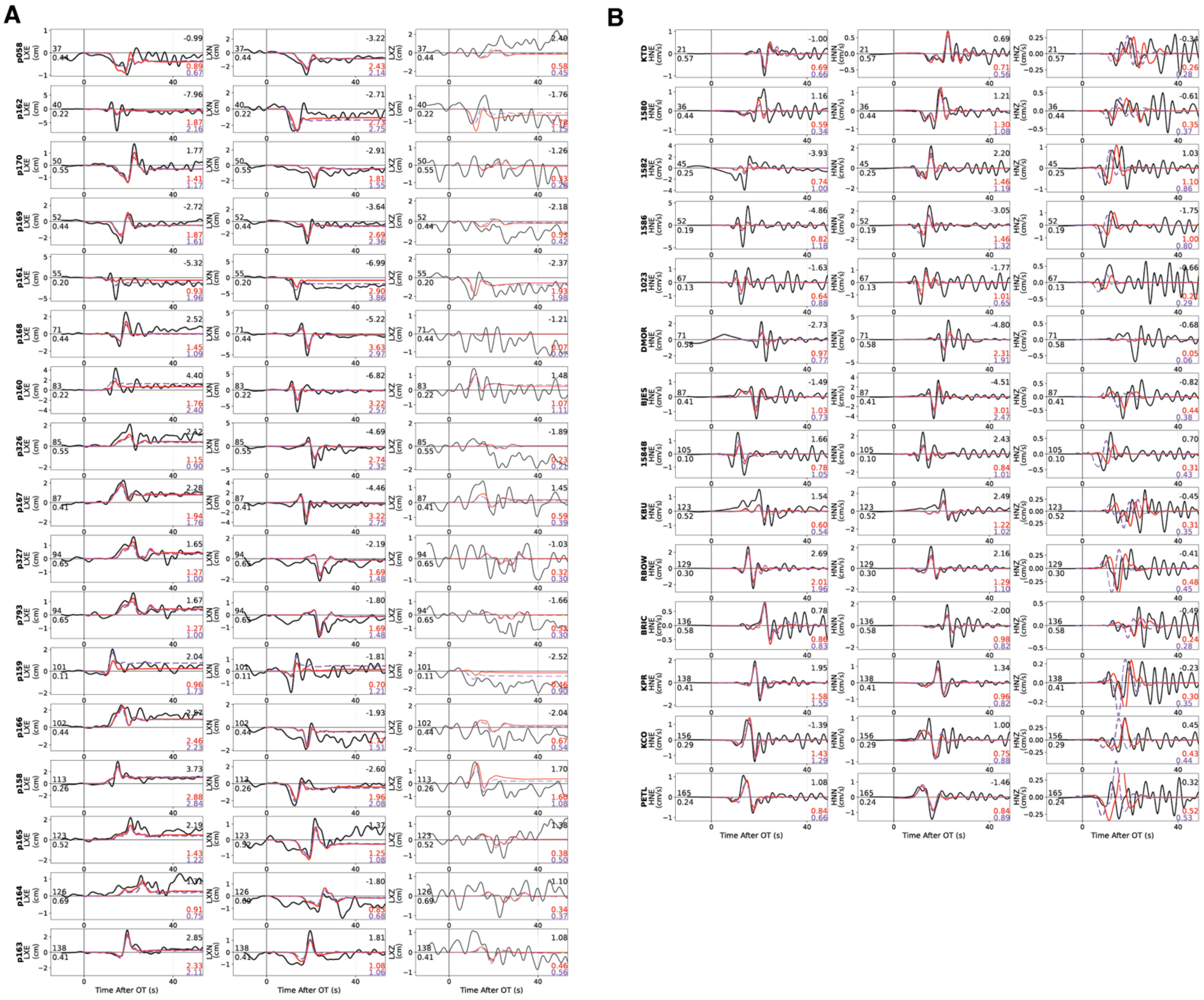

Fig. S5. Finite fault modeling results, comparing depth constrained (red) and unconstrained (purple) models. Observed waveforms (black), depth-constrained (red) and unconstrained (purple) models for **(A)** High-rate GNSS, **(B)** strong motion accelerometer. In the left edge of each plot, the top number denotes the station azimuth while lower value denotes the station distance (in degrees). On the right edge of each plot, the numbers in black, red, and purple denote the maximum amplitude for the observations, depth constrained model, and unconstrained model, respectively.

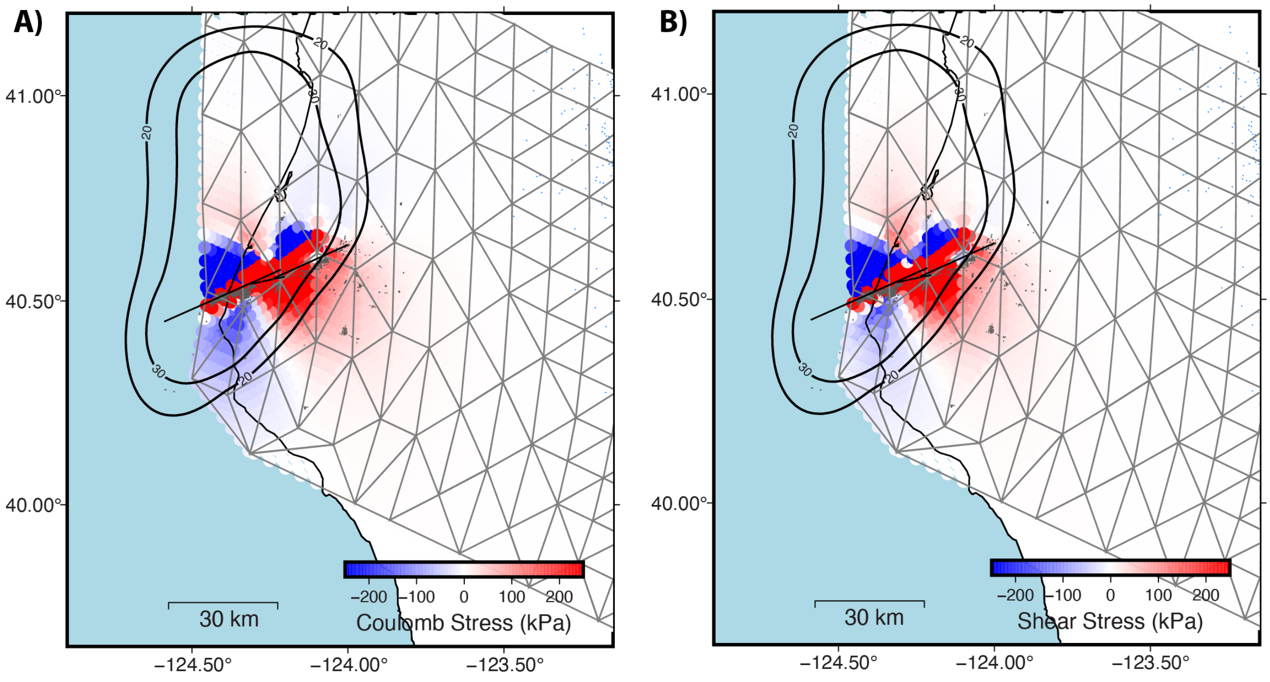

Figure S6. Modeled static stress transfer from depth-constrained coseismic slip model to the subduction interface. (A) Coulomb stress. **(B)** Shear stress. Model assumes coefficient of friction of 0.4, shear modulus of 30 GPa, and Poisson's ratio of 0.25. Rake of all receiver faults was set to 90º (pure reverse). Positive stress is encouraging of subduction. Black contours indicate interseismic locking in mm/year, as in Fig. 4.

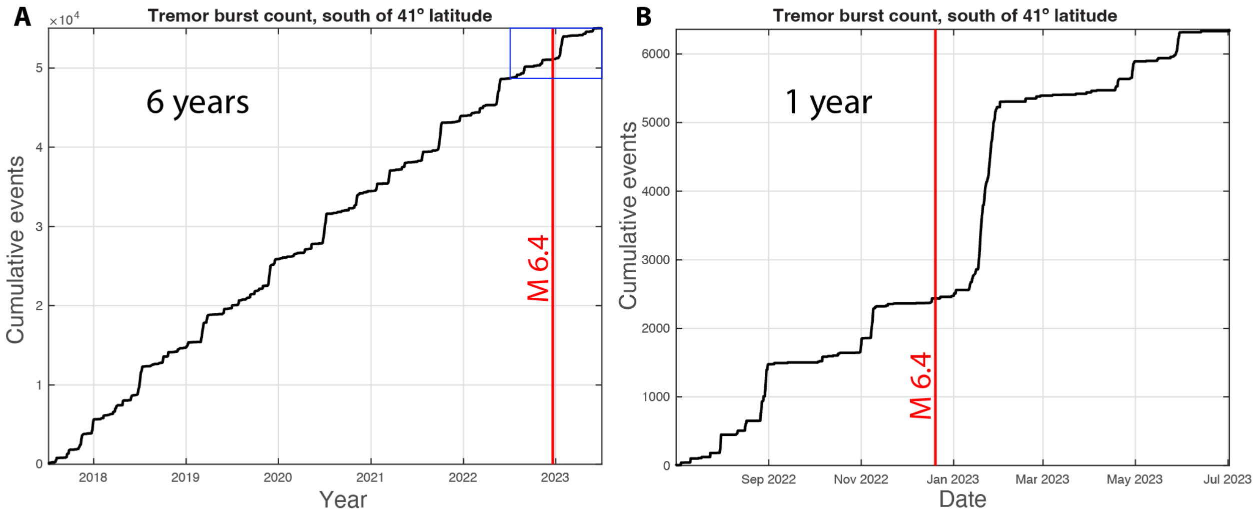

Fig. S7. **Cumulative tremor events with time, for events south of 41° latitude**. **(A)** Six-year time-period. Blue box in upper right shows area of zoomed view in (B). **(B)** One-year time-period. Timing of the *M* 6.4 Ferndale mainshock is shown. (https://pnsn.org/tremor, last accessed July 27, 2023) (*61*).
